# Preterm Neonates Admitted in the Neonatal Intensive Care Unit at a Tertiary Care Centre: A Descriptive Cross-sectional Study

**DOI:** 10.31729/jnma.8126

**Published:** 2023-04-30

**Authors:** Yograj Sharma, Om Krishna Pathak, Bipin Poudel, Asmita Sharma, Ram Prasad Sapkota, Kiran Devkota

**Affiliations:** 1 Department of Pediatrics, Neonatal Intensive Care Unit, Bharatpur Hospital, Bharatpur, Chitwan, Nepal; 2 Bharatpur Hospital Nursing Program, Bharatpur Hospital, Bharatpur, Chitwan, Nepal; 3 Department of Gynaecology and Obstetrics, Bharatpur Hospital, Bharatpur, Chitwan, Nepal

**Keywords:** *morbidity*, *neonatal*, *neonatal intensive care unit*, *premature birth*

## Abstract

**Introduction:**

Preterm birth, one of the leading causes of admissions to the Neonatal intensive care unit, is a major contributor to neonatal morbidity and mortality in developing countries. This study aimed to find out the prevalence of premature neonates admitted to the Neonatal Intensive Care Unit of a tertiary care centre.

**Methods:**

This descriptive cross-sectional study was conducted from clinical records of preterm neonates (born before 37 completed weeks of gestation) admitted in the Neonatal Intensive Care Unit from 16 July 2020 to 14 July 2021. Following ethical approval from the Institutional Review Committee (Reference number: 077/78-018), the patient's clinical characteristics and systemic morbidities were recorded. Convenience sampling was done. Point estimate and 95% Confidence Interval were calculated.

**Results:**

Among 646 admissions, the prevalence of preterm neonates was found to be 147 (22.75%) (19.52-25.98, 95% Confidence Interval). The male: female ratio was 1.53:1. The median gestational age and birth weight were 33 weeks (Range: 24-36 weeks) and 1680 g respectively. A total of 73 (49.65%) delivery was followed by premature rupture of membrane. The morbidity due to respiratory problems was highest at 127 (86.39%), followed by metabolic at 104 (70.74%) and sepsis at 91 (61.90%). The renal system was the least affected 5 (3.40%).

**Conclusions:**

The prevalence of preterm neonates in the neonatal intensive care unit was higher than in other studies done in similar settings.

## INTRODUCTION

Preterm/Premature birth (newborn, alive before 37 completed weeks of pregnancy), a serious perinatal health issue across the globe is a major contributor to neonatal morbidity and mortality and has long-term adverse effects on health.^1-3^ Globally, preterm birth accounts for around 10-6% of live births.^4^ In Nepal, the incidence of preterm births is 9.3%.^5^

Compared to term, preterm newborn have a significantly increased risk of complications and death during the neonatal and post-neonatal period.^6^ Systemic comorbidities associated with prematurity become more evident with increasing duration of stay in the Neonatal Intensive Care Unit (NICU). The prognosis of these newborn has increased during the last three decades due to improvements in neonatal intensive care facilities, yet rates vary widely across different centres.^7^

This study aimed to find out the prevalence of premature neonates admitted in the neonatal intensive care unit of a tertiary care centre.

## METHODS

This descriptive cross-sectional study was conducted in the Neonatal Intensive Care Unit (NICU) of Bharatpur Hospital, Chitwan, Nepal for a period of one year from 16 July 2020 to 14 July 2021. Data collection was started after receiving ethical approval from the Institutional Review Committee of Bharatpur Hospital (Reference number: 077/78-018). After informed consent from the parents or immediate caretaker, all newborn admitted to the NICU who were born before 37 completed weeks of gestation were enrolled in the study. Both in and out-born were included. Newborn previously admitted to another institute for more than 72 hours before transfer to our institute, who required referral for surgical problems and whose parents denied consent were excluded. The sample size was calculated by using the following formula:


$$\begin{array}{ll} \text{n} & ={\text{Z}}^{2}\times \frac{\text{p}\times \text{q}}{{\text{e}}^{\text{2}}}\\ & =1.96^{2}\times \frac{0.165\times 0.835}{0.03^{2}}\\ & =589 \end{array}$$

Where,

n = minimum required sample size
Z = 1.96 at 95% Confidence interval (CI)
p = prevalence of preterm neonates in NICU from the previous study, 16.48%^8^
q = 1-p,
e = margin of error, 3%

The calculated sample size 589, However sample size of 646 was included in the study.

Patient particulars including weight at admission, gestational age, place and mode of delivery, need of resuscitation at birth etc. and maternal factors including age, gravida and parity, causes of preterm birth, pregnancy-associated complications, use of antenatal steroids etc.were recorded at admission to the NICU. Patients were followed till transfer/discharge or referral from the unit or till death. Systemic morbidities encountered during treatment in the NICU were followed and recorded.

Gestational age was ascertained from the mother's first day of the last menstrual period (LMP). If the mother was uncertain of the LMP, an early trimester USG scan and maturity assessment using Modified Ballard Score were considered.^9^ Clinical diagnoses were made based on the standard guidelines being used in the Unit. Relevant laboratory investigations and imaging techniques were used whenever necessary. Sepsis was diagnosed on clinical grounds along with C-reactive protein (CRP), complete blood count (CBC), positive blood culture, and cerebrospinal fluid (CSF) examination. Respiratory distress syndrome (RDS), meconium aspiration syndrome (MAS) and pneumonia were diagnosed by X-ray and ultrasound of the chest along with relevant laboratory and clinical findings. Congenital heart disease was confirmed by Echocardiography. Hypoglycemia was defined as a blood sugar level <45 mg/dl and hypocalcemia as total serum calcium <7 mg/dl.^10^ Gestational age and hour-specific bilirubin thresholds (adopted from AAP & NICE guidelines) were used to determine the need for phototherapy for Hyperbilirubinemia.^11,12^

Data was entered in Microsoft Excel version 2010 and analysed using IBM SPSS Statistics version 20.0. Point estimate and 95% CI were calculated.

## RESULTS

Among 646 total admissions in the NICU, the prevalence of preterm neonates was found to be 147 (22.75%) (19.52-25.98, 95% CI). The majority of mothers of the preterm neonates were from Province 388 (59.86%) and a total of 128 (87.07%) had their deliveries in a hospital setting (Table 1).

**Table 1:** Demographic profiles (n= 147).

Variables	n (%)
**Provinces**	
Province 2	9 (6.12)
Province 3	88 (59.86)
Province 4	50 (34.01)
**Place of delivery**	
Hospital	128 (87.07)
Ambulance (on the way to the hospital)	8 (5.44)
Health post	7 (4.76)
Home	4 (2.72)
Inborn	111 (75.51)
Outborn	36 (24.48)
Referred from another centre	43 (29.25)

The mean age of the mothers was 24.50±5.30 years, with a median gestational age of 33 weeks at the time of delivery (Range: 24-36 weeks). More than half of the mothers 80 (54.42%) were multigravida. Only about 70 (48.27%) mothers had ≥4 Antenatal care (ANC) visits. A total of 56 (38.09%) underwent caesarean section (CS), the commonest indication being previous CS 21 (37.50), followed by multiple pregnancies 10 (17.85) (Table 2).

**Table 2:** Maternal and obstetric characteristics (n = 147).

Maternal characteristics	n (%)
**Gravida**	
Primi	63 (42.85)
Multi (≥2)	80 (54.42)
Grand multi (>5)	4 (2.72)
ANC visits	145 (98.63)
<4	75 (51.72)
≥4	70 (48.27)
**Obstetric characteristics**	
CS	56 (38.09)
vaginal delivery	91 (61.90)
**Indication for CS**	
Previous CS	21 (37.50)
Multiple pregnancies	10 (17.85)
Oligohydramnios	6 (10.71)
Breech presentation	6 (10.71)
Antepartum haemorrhage (APH)	6 (10.71)
Non-progress of labour (NPOL)	3 (5.35)
Foetal distress	2 (3.57)
Cord prolapse	1 (1.78)
Transverse lie	1 (1.78)

Out of total preterm neonates admitted to NICU, 89 (60.54%) were male and 58 (39.45%) were female.

The median age (in hours) and weight at the admission of the preterm neonates was 1 hr (Range: 0-72 hr) and 1680 gm (Range: 625-3125 gm) respectively. The most frequent cause of preterm delivery was premature rupture of membrane (PROM) 73 (49.65%), followed by spontaneous onset of labour 44 (29.93%) (Table 3).

**Table 3:** Underlying condition of preterm birth (n= 147).

Conditions	n (%)
PROM	73 (49.65)
Spontaneous onset of labour	44 (29.93)
Multiple pregnancies	10 (6.80)
Antepartum haemorrhage (APH)	12 (8.16)
Pregnancy induced hypertension (PIH)	6 (4.08)
Fetal distress	2 (1.36)

The prevalence of respiratory morbidity was the highest 127 (86.39%), followed by metabolic 104 (70.74%) and infectious morbidity 91 (61.90%). Respiratory Distress Syndrome (RDS) and Apnea of prematurity (AOP) were the most common respiratory morbidities. The mean duration of NICU stay was 8.57±7.55 days. The in-hospital mortality rate was 17 (11.56%) (Table 4).

**Table 4:** Description of systemic morbidities in the preterm neonates (n = 147).

Variables	n (%)
Respiratory morbidity	127 (86.39)
RDS	96 (65.30)
Apnea	41 (27.89)
Transient tachypnea of the newborn (TTN)	20 (13.60)
Pneumonia	19 (12.92)
Persistent pulmonary hypertension of the newborn (PPHN)	3 (2.04)
Air leak	2 (1.36)
Meconium aspiration syndrome (MAS)	1 (0.68)
Bronchopulmonary dysplasia (BPD)	1 (0.68)
Metabolic morbidity	104 (70.74)
Hypoglycemia	42 (28.57)
Temperature instability	16 (10.88)
Hypocalcemia	18 (12.24)
Hyperbilirubinemia requiring phototherapy	75 (51.02)
Hyperglycemia	2 (1.36)
Infectious morbidity	91 (61.90)
Presumed/Clinical sepsis	85 (57.82)
Culture proven sepsis	6 (4.08)
**Sepsis type**	
Early onset	64 (43.53)
Late-onset	27 (18.36)
**Cardiovascular morbidity**	49 (33.33)
Patent ductus arteriosus (PDA)	15 (10.20)
Atrial septal defect (ASD)	12 (8.16)
Ventricular septal defect (VSD)	3 (2.04)
Shock requiring inotrope support	30 (20.40)
**Gastrointestinal morbidity**	41 (27.89)
Feeding intolerance	23 (15.64)
NEC	21 (14.28)
Others (Gastrointestinal bleed, perforation)	3 (2.04)
**Haematological morbidity**	41 (27.89)
Anaemia requiring blood transfusion	28 (19.04)
Coagulopathy	9 (6.12)
Polycythemia	5 (3.40)
Neurological morbidity	36 (24.48)
Intraventricular haemorrhage (IVH)	12 (8.16)
Seizures	12 (8.16)
Birth asphyxia	18 (12.24)
Hydrocephalus	1 (0.68)
**Renal morbidity**	5 (3.40)

## DISCUSSION

In our study, the prevalence of preterm neonates was found to be 147 (22.75%). It is higher than the prevalence reported by similar studies from Nepal and Pakistan (16.4-20.3%).^8,13^ A total of 147 preterm neonates (born before 37 weeks of gestation) fulfilling inclusion criteria were enrolled. The average gestational age of the study population was 33 weeks, which is consistent with the study population described in a similar study from Pakistan (32.4 weeks) and lower than similar other studies from Nepal and India (>34 weeks).^13-15^ There was male predominance in this study (60.5%) which is consistent with similar other studies from Nepal (56-74.5%) which might suggest that the male gender in Nepalese society gets more attention on the part of caregivers and is brought to seek health services.^8,14^

The median birth weight of the study population was 1700 gm (range 675-3200 gm) which is lower than the study from BPKIHS, Dharan (181 gm) and Pakistan (1795 gm) but higher than a similar study from Lumbini Medical College, Nepal (1188.9 gm).^8,13,16^

The most common maternal risk factor for preterm delivery was PROM (49.6%) followed by spontaneous onset of labour (30%) and multiple pregnancies (6.8%) which is consistent with the study done at Lumbini.^8^ However similar other studies from Nepal and India reported PIH as a more common cause of preterm birth (12.9-26%).^14-16^

In this study, 86.4% of all preterm infants had respiratory morbidities of which RDS, apnea of prematurity, and pneumonia were the commonest comprising 65.3%, 27.9%, and 12.9% respectively. This is consistent with the study conducted at TUTH, Nepal which also reported RDS as the most common morbidity among preterm infants.^14^ However, the incidence of RDS and apnea of prematurity in the present study is greater than the similar studies from Nepal (32% and 7%), and India (38.3% and 9%).^14,15^ This might be due to the lower mean gestational age of our study population.

At least one form of metabolic complication (hypoglycaemia, hypocalcemia, hyperbilirubinemia or hyperglycemia) was encountered in 70.7% of preterm infants. Among these, hyperbilirubinemia (51%) and hypoglycaemia (28.6%) were the commonest. This finding is consistent with similar studies from Nepal and other developing countries.^8,13-15^

Sepsis was the second most common individual morbidity after RDS in this study occurring in almost two third of the cases, out of which only 6.5% had positive blood cultures. Among neonates who had sepsis, the majority (70%) were early onset. The occurrence of sepsis in the present study was higher as compared to similar studies from other centres of Nepal (37-40%) and Pakistan (28.6%).^8,13,14^ Higher incidence of sepsis may be because of the inclusion of cases with clinical sepsis. Additionally, the unit receives a significant number of outborn neonates with sepsis referred from other centres.

Among other systemic morbidities, cardiovascular, gastrointestinal and neurological morbidities were encountered in 33%, 27.9% and 24.5% of the cases respectively. Around 20% of the neonates had shock which was lower in comparison to a study done in a similar population at BPKIHS, Dharan (43%).^16^ This study included cases of fluid refractory shock requiring inotropic support only, which could account for the lower incidence. PDA was the most common congenital heart defect found in the study population which is consistent with the finding in similar other studies.^14,16^

Feed intolerance (15.6%) and NEC (14.3%) were the commonest GI morbidities consistent with similar other studies.^13,15^ The incidence of NEC in this study is similar to the study from Pakistan (15.5%) but is higher than studies done at other centres in Nepal and India (2.2%-4%).^8,13-15^ The higher incidence of NEC may be due to the lower mean gestational age of our study population.

Birth asphyxia was the most common neurological morbidity in the present study, accounting for about 12% of all cases followed by seizures and IVH each accounting for 8%. A similar study from India also reported birth asphyxia and seizures in 11% of their cases.^15^ However, the incidence of birth asphyxia in this study is higher compared to other studies (3.5%-9.7%).^8,13,14^ This might be because this unit receives a significant number of referred and complicated cases from birthing centres and hospitals of nearby districts.

Our study was based on data from NICU at a single tertiary care facility with a small sample size within a limited time frame. So, it cannot be generalised to the broader prospect. Furthermore, since this is a descriptive study, we were unable to find associations between the variables and neonatal morbidities.

## CONCLUSIONS

The prevalence of preterm neonates in the Neonatal Intensive Care Unit was higher than in other studies done in similar settings.
